# Bioinspired Engineering towards Tailoring Advanced Lignin/Rubber Elastomers

**DOI:** 10.3390/polym10091033

**Published:** 2018-09-18

**Authors:** Haixu Wang, Weifeng Liu, Jinhao Huang, Dongjie Yang, Xueqing Qiu

**Affiliations:** 1 School of Chemistry and Chemical Engineering, South China University of Technology, Guangzhou 510640, China; haixuwang123@163.com (H.W.); cejhhuang@scut.edu.cn (J.H.); cedjyang@scut.edu.cn (D.Y.); 2 State Key Laboratory of Pulp and Paper Engineering, South China University of Technology, Guangzhou 510640, China

**Keywords:** lignin, nitrile rubber elastomers, dual-crosslinking network, coordination sacrificial bonds, energy dissipation

## Abstract

The pursuit of high volume and high value-added applications for lignin has been a long-term challenge. In this work, inspired by the energy sacrificial mechanism from biological materials, we developed high-performance lignin/carbon black (CB)/nitrile rubber (NBR) elastomers by constructing a dual-crosslinking network consisting of sulfur covalent bonds and dynamic coordination sacrificial bonds. Lignin was not only used for the substitution of half mass of CB in the NBR elastomer but also served as natural ligands for the Zn-based coordination bonds, providing a significant synergistic coordination enhancement effect. The mechanical performance of the elastomers can be easily manipulated by adjusting the proportion of non-permanent coordination bonds and permanent covalent bonds. Lignin/CB/NBR elastomers with a higher strength and modulus than CB-filled elastomers were obtained while maintaining excellent elasticity. The thermal stability and the high-temperature oil resistance of NBR elastomers were also improved by incorporation of lignin and metal coordination bonds. Overall, this work inspires a new solution for the design of high-performance lignin/rubber elastomers with a high lignin loading content.

## 1. Introduction

Increasing concerns over global warming and resource scarcity have driven a rising focus on the development of green materials from biorenewable alternatives. The efficient utilization of biomass resources and the innovation of biomimetic studies provide us with enormous possibilities for constructing sustainable materials. Recently, the energy sacrificial bonds have been demonstrated as a key mechanism in determining the outstanding properties of some strong and tough biomaterials such as the spider silk and mussel byssus [1]. The sacrificial bonds can undergo reversible rupture and reconstruction before covalent bonds break and thus effectively dissipate mechanical energy under loading. They can be reversible dynamic non-covalent bonds formed by hydrogen bonds, ionic bonds and/or coordination bonds [2]. When the energy sacrificial bonds are applied in hydrogels, the strength and toughness can be substantially improved [3,4]. Costantino [5] reported that the strength and toughness of polyacrylate elastomers were greatly improved by building a multi-level crosslinked network containing energy sacrificial bonds. Guo [6] introduced Zn^2+^-pyridine coordination bonds into the sulfur-crosslinked styrene-butadiene-vinylpridine rubber (VPR) to construct a dual-crosslink network. The strength and the fracture energy of VPR were increased by seven and five times, respectively. Moreover, the presence of sacrificial bonds facilitates elimination of the stress concentration and promotes the strain-induced-crystallization [7]. When the sacrificial bonds are incorporated into the interphase between rubber and fillers, the interfacial interactions can be enhanced, promoting the filler dispersion and stress transmission [8]. As well known, rubber products are usually manufactured by compounding with a large quantity of reinforcing agents, due to the poor mechanical properties of rubber itself. Up until now, carbon black (CB) was still the most commonly used reinforcing agent in the rubber industry. However, its production is energy consumptive and highly dependent on fossil resources with a high cost [9]. It is essential to find a cheap, readily available, renewable and environmentally friendly alternative.

Lignin, a primary constituent of all higher plants, is considered to be the second most abundant renewable bio-resource. Industrial lignin is a major by-product of the pulping and papermaking industries with an annual global output of more than 50 megatons [10]. However, over 95% of industrial lignin is either burned as cheap fuel or discharged as wastewater, causing a serious waste of resources and environmental pollution [10]. Additional high volume and high value-added applications for lignin are strongly demanded by world market.

The pursuit of high-performance lignin-based rubber elastomers has been a long-term challenge and has attracted hot attention in both the industrial and academic community. Using lignin as a replacement for the conventional rubber filler is highly competitive due to its natural abundance, cost competitiveness, and more importantly, excellent UV-resistant performance [11]. However, the reinforcement effect of lignin is not as good as CB attributing to the poor dispersion ability of lignin in rubber (large lignin particle size) and the poor interfacial compatibility between lignin and rubber matrix [12]. For decades, people have tried many methods to improve the reinforcement effect of lignin in rubber [13,14,15,16,17,18]. But the reported methods are either complex or cost-intensive, making lignin less competitive compared with conventional rubber fillers. The reinforcement effect of lignin is still much poorer than that of CB.

Lignin is a three-dimensional natural polymer rich in oxygen-containing polar groups, such as the phenolic hydroxyl groups, aliphatic hydroxyl groups, and carboxyl groups, which can efficiently function as natural ligands for the coordination bonds. Therefore, we were inspired by the idea to introduce the coordination sacrificial bonds into the interphase between lignin and a rubber matrix to make elastomers with a high strength and toughness.

In this paper, industrial alkali lignin was used to reinforce nitrile rubber (NBR). The alkali lignin used in this work came from black liquors obtained by sodium hydroxide treatment of wheat straw, also known as the alkali pulping process, which is currently the major industrial pulping process. The study on the alkali lignin as a reinforcer in NBR is of great importance to the comprehensive utilization of black liquors from alkali pulping plants. Here, lignin was not only used as the reinforcing filler for the substitution of half the mass of CB in NBR but also served as natural ligands for the Zn-based coordination bonds. The coordination sacrificial bonds were incorporated in the interphase between lignin and a rubber matrix. A dual-crosslinking network consisting of the sulfur covalent bonds and coordination sacrificial bonds was constructed. The tensile strength and elastic modulus of lignin/CB/NBR elastomers were significantly increased in comparison with the precursor without coordination sacrificial bonds. The enhanced performance originates from the synergistic coordination enhancement effect of lignin, which allows higher energy dissipation through the dynamic rupture of Zn-based coordination bonds. The effects of coordination bonds on the performance of rubber elastomers were studied in detail. Through the implementation of this work, a novel method for the preparation of high-performance lignin-based rubber elastomers was established.

## 2. Materials and Methods

### 2.1. Materials

NBR N220S (acrylonitrile content 41 wt %) was purchased from Japan Synthetic Rubber Corporation (Osaka, Japan). Industrial alkali lignin (*M*_w_ = 5900 g/mol and *Đ* = 2.7, purity ~85%) was provided by Xiangjiang Paper Co., Ltd. (Hunan, China) and used without further purification or chemical modification. The phenolic hydroxyl content of the alkali lignin was ~2.24 mmol/g, the alcohol hydroxyl content was ~1.11 mmol/g, and the carboxy group content was ~1.55 mmol/g.

Carbon Black (N660) was purchased from Guangzhou Liben Rubber Material Trading Co., Ltd. (Guangzhou, China). ZnCl_2_ (AR grade) was provided by the Tianjin Damao Chemical Reagent company (Tianjin, China). Toluene (AR grade) was provided by the Guangzhou Chemical Reagent company (Guangzhou, China). Hypoid gear oil (API grade GL-3, viscosity grade 75W-80) was from Jiangsu Anda Lubricating Oil Co., Ltd. (Zhenjiang, China). All other rubber ingredients of analytical grade, such as zinc oxide (ZnO), stearic acid (SA), sulfur (S), *N*-cyclohexyl-2-benzothiazolylsulfenamide (CZ) and 2,2-dithiobisbenzothiazole (DM), were purchased from Shanghai Aladdin Bio-Chem Technology Co., Ltd. (Shanghai, China). All chemicals were used as received.

### 2.2. Sample Preparation

The lignin/CB/NBR elastomers were prepared through a banbury mixer of 100 mL (Guangdong Lina Co., Ltd., Dongguan, China) with the formulations listed in Table 1. NBR was firstly mixed with lignin and CB at the speed of 50 rpm at room temperature for 10 min. Then, ZnCl_2_ was added and mixed for 5 min, followed by adding all other rubber ingredients (ZnO, SA, S, CZ, DM) and continued for another 5 min. The blend was then removed from the mixer and pressed into a sheet on an open twin-roll mill. After aging at room temperature for 24 h, the sample was finally press-cured into a 1 mm thick sheet at 160 °C for the optimum cure time (*T*_90_). The preparation of lignin/NBR elastomers and CB/NBR elastomers was similar to that of lignin/CB/NBR elastomers. LxCyZmSn is the abbreviated sample name with “L” standing for lignin, “C” for CB, “Z” for ZnCl_2_, “S” for sulfur, and the numbers *x*, *y*, *m* and *n* representing the corresponding weight parts relative to 100 parts of NBR.

### 2.3.Characterization

Fourier transform infrared spectroscopy (FTIR) spectra were obtained on a Bruker Vertex 70 FTIR spectrometer (Bruker, Ettlingen, Germany). The curing characteristics were analyzed by U-CAN UR-2010SD-A vulcameter (U-CAN dynatex, Taipei, Taiwan) at 160 °C. Scanning electron microscopy (SEM) analysis was conducted on a Hitachi UHR FE-SEM SU8220 instrument (Hitachi, Tokyo, Japan) to study the fracture surface of the samples. The Shore A hardness was measured using a LX-A durometer (Kunshan Creator Testing Instrument Co., Ltd., Kunshan, China) according to GB/T531-1999.

Tensile tests were carried out on an electronic universal testing machine (MTS Systems Co., Ltd., Shenzhen, China) according to the standard GB/T528-2009 at an elongation speed of 200 mm/min at 25 °C. The elastic recovery was calculated using Equation (1):

(1) $$\mathrm{Elastic}\;\mathrm{recovery}=\frac{l_{1}-l_{2}}{l_{1}-l_{0}}\times 100\%,$$

where *l*_0_ is the initial sample length, *l*_1_ is the maximum stretched length at break, *l*_2_ is the recovered length after break.

For the hysteresis test, the samples were stretched to a fixed strain of 250% and returned to the initial position at 25 °C for the first cycle. The stretching and returning speed was 100 mm/min. Then, the samples stayed for a respective period of time (0, 60, 120, 300, 600, 1800, 3600, 7200 s) to restore the samples before the next cycle. Then the samples were warmed for 40 min at 80 °C for a quick recovery and cooled to room temperature, followed by the last cycle. The loading energy for the first stretching, *W*_0_, was defined as the area integral of the first stretching stress-strain curve. *W*_1_ is the dissipation energy of the first hysteresis loop and *W*_2_ is the dissipation energy of the second hysteresis loop. Δ*W* is defined as the hysteresis difference (*W*_1_ − *W*_2_). *W*_h_ resembles the dissipation energy of the last hysteresis loop after heating.

For the stress relaxation tests, the samples were stretched to a fixed strain of 200% with a stretching speed of 100 mm/min at 25 °C. Then the variation of stress was recorded for 3600 s with constant strain.

Dynamic mechanical analysis (DMA) was performed on a Q800 dynamic thermal mechanical analyzer (TA instrument, New Castle, DE, USA). The samples were measured in tensile mode at a dynamic strain of 0.5% with a preload of 0.01 N and a frequency of 1 Hz. The temperature scan range was from −90 to 100 °C and the heating rate was 3 °C/min.

Thermal behaviors were measured by differential scanning calorimetry (DSC) using a Netzsch 214 instrument (Netzsch, Bavaria, Germany) under a flowing nitrogen atmosphere. Firstly, the samples were heated to 200 °C and kept for 3 min to remove the heat history. Then the samples were cooled to −70 °C and kept for 3 min and reheated up to 200 °C. The heating and cooling rate was 10 °C/min. The second heating curves were used for the determination of glass transition temperature *T*_g_.

Thermal gravimetric analysis (TGA) was conducted in a Netzsch STA 449F3 thermogravimetric analyzer (Netzsch, Bavaria, Germany) over a temperature range from 25 to 600 °C at a scan rate of 10 °C/min under a nitrogen atmosphere. An oil resistance measurement was conducted by immersing the rubber specimens in hypoid gear oil at 100 °C for 72 h. Before testing, the specimens were vacuum dried at 100 °C for 24 h. The mass variation at different times was recorded during the oil immersion.

The crosslinking density of the cured samples was evaluated by the swelling equilibration. Firstly, the samples were dried to constant initial weight (*m*_1_). Then the samples were immersed into toluene at 25 °C for 72 h. Finally, the samples were taken out from toluene and the swollen weight (*m*_2_) of the samples was recorded. The crosslinking density (*μ*) was calculated with the Flory-Rehner model (Equation (2)) [19]:

(2) $$\mu =\frac{-\left[ln\left(1-\nu \right)+\nu +\chi \nu^{2}\right]}{V\left(\nu^{\frac{1}{3}}-\frac{\nu }{2}\right)},$$

where ν is the volume fraction of polymer in a swollen gel at equilibrium and *V* is the molar volume of toluene (*V* = 105.9 cm^3^/mol). The interaction parameter (χ) between NBR and toluene was 0.691 according to the Bristow-Watson equation [20]. ν is calculated by the Equation (3):

(3) $$\nu =\frac{\left(m_{1}-m_{1}\phi \right)/\rho_{1}}{\left(m_{1}-m_{1}\phi \right)/\rho_{1}+\left(m_{2}-m_{1}\right)/\rho_{2}},$$

where ϕ is the mass fraction of filler and $\rho_{1}$ and $\rho_{2}$ are the densities of NBR and toluene, respectively.

## 3. Results

### 3.1. Introduction of Zn-Based Sacrificial Bonds into Lignin/CB/NBR Elastomers

**Figure 1:**
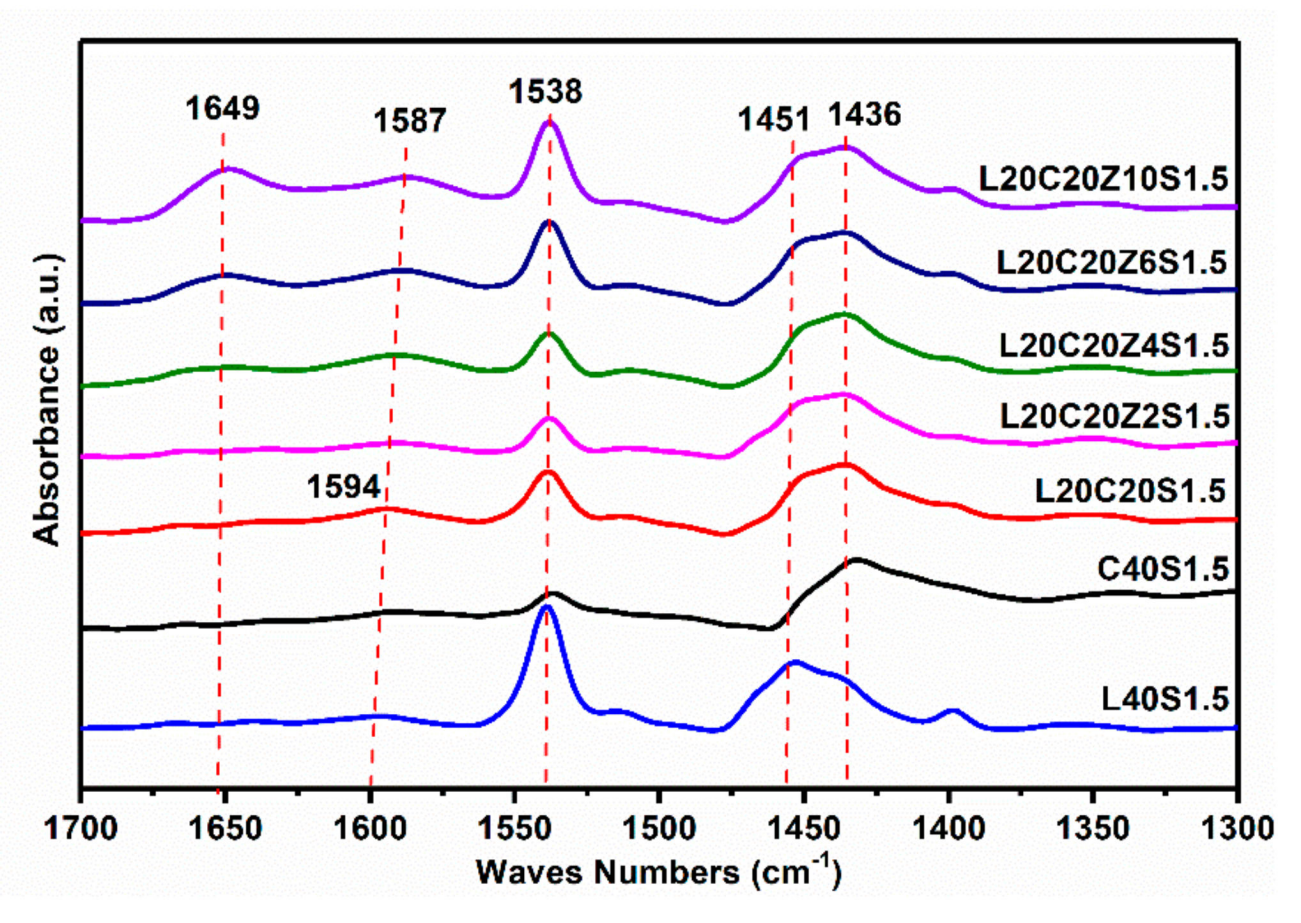
FTIR spectra of NBR elastomers with different contents of Zn^2+^.

**Scheme 1:**
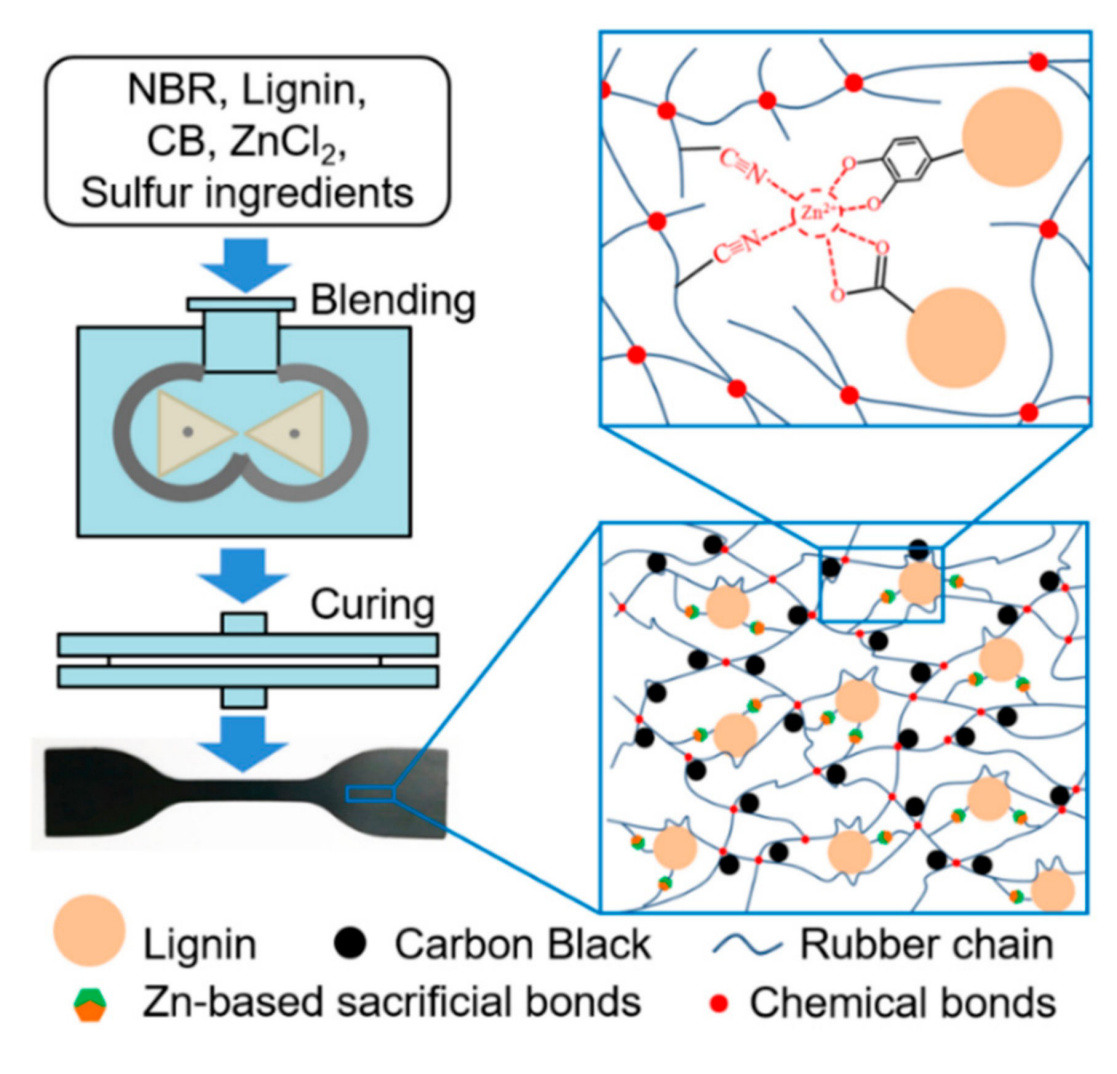
Constructing mechanism of lignin/CB/NBR elastomers with Zn-based coordination sacrificial bonds.

**Scheme 2:**
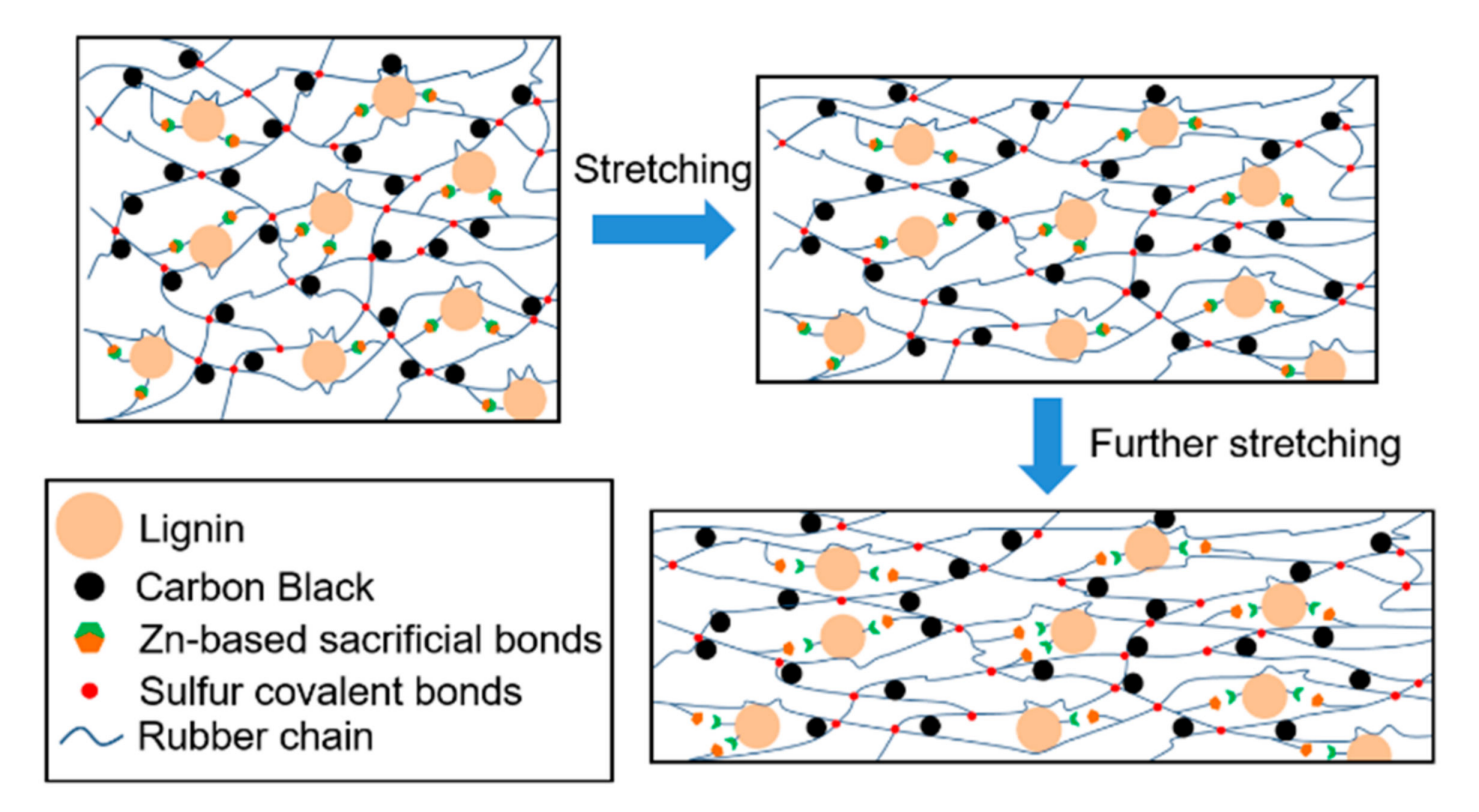
Proposed deformation mechanism for the lignin/CB/NBR elastomers.

Inspired by the energy sacrificial mechanism commonly existing in biological materials, the coordination sacrificial bonds were introduced into the lignin/CB/NBR elastomers through simply adding ZnCl_2_ during the compounding process. The preparation process and schematic rubber structure are illustrated in Scheme 1. The Cl^−^ anions still existed in the composite system but they did not participate in the formation of metal coordination bonds within lignin/CB/NBR. Therefore the Cl^−^ anions are not shown in Scheme 1 (and Scheme 2). The formation of metal coordination bonds in the lignin/CB/NBR elastomers was verified by FTIR spectra, as shown in Figure 1. The absorption peaks at 1451 and 1436 cm^−1^ were assigned to the C–H stretching deformation in methyl or methylene groups and aromatic rings. Compared with the sample C40S1.5, L40S1.5 exhibited a stronger absorption at 1538 cm^−1^, which was assigned to the asymmetric stretching of the metal carboxylate ion formed by the complexation of a zinc cation with carboxyl groups in lignin [21]. Here the zinc cation came from the reaction of zinc oxide and stearic acid that were used as the accelerator in rubber formulations. After introducing additional ZnCl_2_ into the lignin/CB/NBR elastomers, the peak intensity of 1538 cm^−1^ increased with the Zn^2+^ content (as seen from the samples L20C20S1.5 to L20C20Z10S1.5). The characteristic peak at 1594 cm^−1^ assigned to the aromatic skeletal vibrations in lignin shifted to 1587 cm^−1^ with an increasing content of Zn^2+^, indicating the possible coordination of Zn^2+^ with the phenoxy groups on the aromatic rings of lignin. It might also be induced by the asymmetric stretching of the carboxylate coordinated with Zn^2+^ [21]. At a higher Zn^2+^ content, the peak at 1649 cm^−1^ intensified, which further confirmed the complexation of Zn^2+^ with carboxylate groups [17].

### 3.2. Curing Characteristics of Lignin/CB/NBR Elastomers

**Figure 2:**
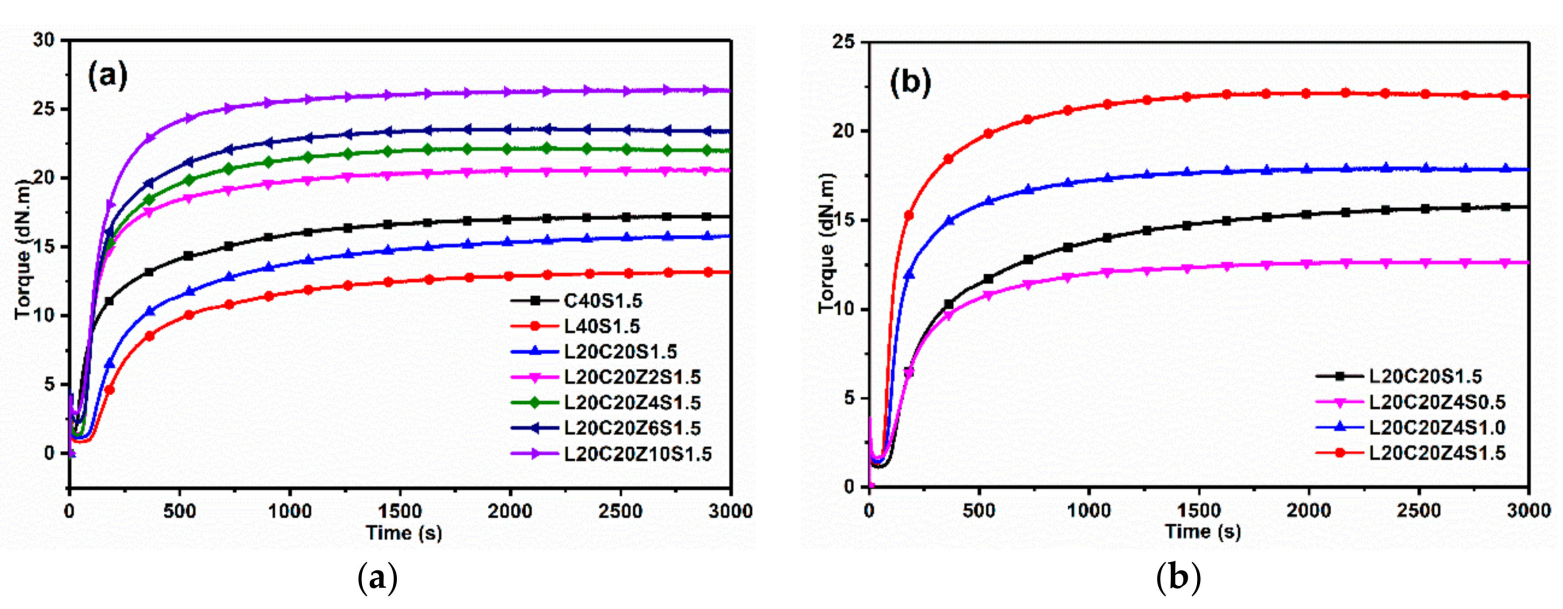
Curing curves of NBR elastomers with (**a**) different contents of Zn^2+^ and (**b**) different contents of S.

The curing characteristics of NBR elastomers are shown in Figure 2 and Table 2. When CB was gradually replaced by lignin, the optimum cure time *T*_90_ and the scorch time *T*_S_ increased, the curing rate index CRI and the crosslinking density *μ* decreased, suggesting that lignin delayed the vulcanization of NBR. This was attributed to the radical scavenging effect of hindered phenol groups in lignin [22]. The values of minimum torque *M*_L_, maximum torque *M*_H_ and Δ*M* (*M*_H_ − *M*_L_) all decreased with the increasing lignin loading, indicating a worse interfacial adhesion between lignin and NBR segments than that between CB and NBR.

When Zn^2+^ was added into the lignin/CB/NBR elastomers, the values of *M*_L_, *M*_H_ and Δ*M* significantly increased. As *M*_L_ mainly reflected the intensity of physical crosslinking before vulcanization [23], it increased obviously with the Zn^2+^ content, suggesting stronger physical crosslinking at higher Zn^2+^ content. In presence of Zn^2+^, the rubber elastomer was composed of chemical crosslinking vulcanized by sulfur and physical crosslinking constructed by the Zn^2+^-based coordination bonds. The introduction of Zn^2+^ also promoted the vulcanization process, as verified by the increased CRI value at a higher Zn^2+^ content. The curing characteristics of NBR elastomers L0C0S1.5 and L0C0Z10S1.5 without fillers also showed the same regularities, as shown in Figure S1 and Table S1 (Supplementary Materials), further proving the strong physical crosslinking formed by coordination interactions in the rubber matrix. Simultaneously, increasing the Zn^2+^ content resulted in an increment of crosslinking density, as more bound rubber [23] was generated under a relatively strong coordination interaction in the interphase between lignin and rubber and also in the rubber matrix.

When the sulfur content decreased, the values of *T*_90_ and *T*_S_ increased and the CRI value decreased. Meanwhile, the values of *M*_H_ and Δ*M* decreased obviously, due to the evidently reduced crosslinking density. Through the adjustment of physical and chemical crosslinking, elastomers with different crosslinking densities were prepared. The FTIR analysis, the curing characteristics and the evaluation of crosslinking density all demonstrated the successful incorporation of Zn-based coordination bonds into the lignin/CB/NBR elastomers.

### 3.3. Mechanical Performance of Lignin/CB/NBR Elastomers

**Figure 3:**
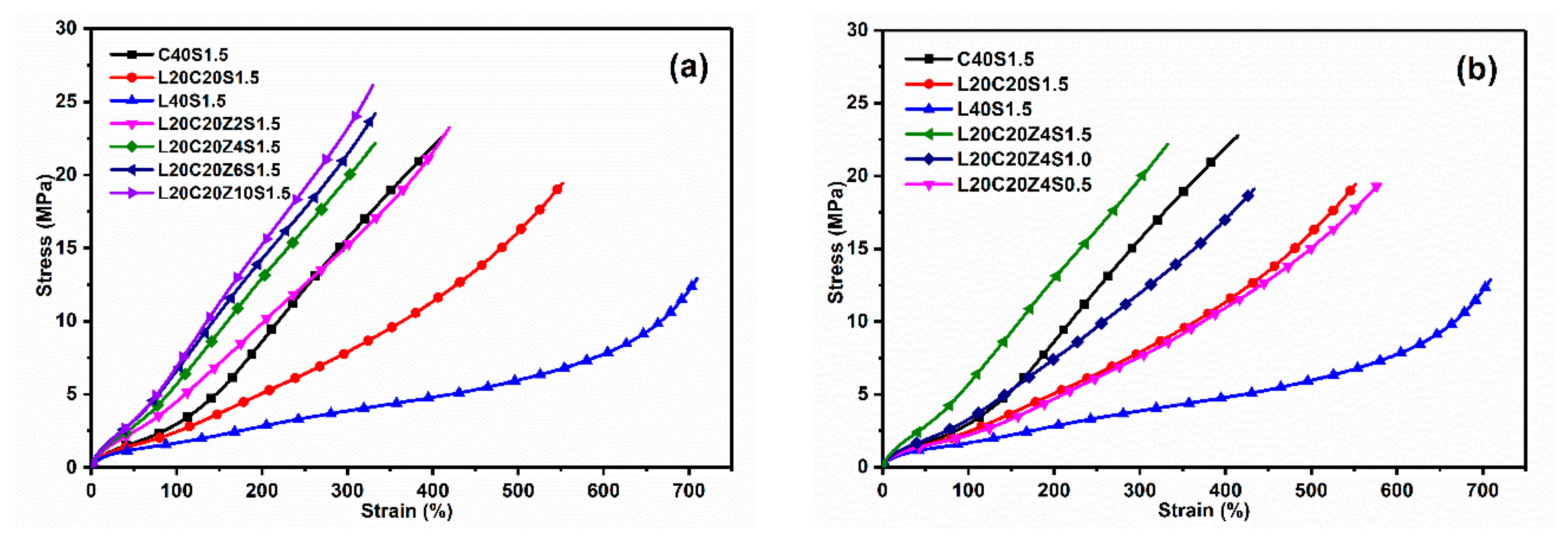
The engineering stress-strain curves of NBR elastomers with different contents of (**a**) Zn^2+^ and (**b**) S.

The tensile stress-strain curves versus the Zn^2+^ and S content are depicted in Figure 3. The characteristic data are shown in Table 3. All the rubber elastomers exhibited excellent elasticity, as proved by the near 100% elastic recovery after break, irrespective of the Zn^2+^ or S content. The tensile strength, Young modulus, toughness (fracture energy dissipation) and hardness of elastomers reduced significantly when CB was substituted by lignin, verifying a worse rubber reinforcement of lignin compared with CB. For instance, the strength at 300% strain for L40S1.5 was about 3.9 MPa, which was only a quarter of that for C40S1.5 (15.7 MPa).

When Zn^2+^ was added into the lignin/CB/NBR elastomers, the tensile strength, Young modulus and hardness substantially increased, benefiting from the coordination sacrificial bonds formed in the elastomers. With the same content of the vulcanizing agent, introducing a small fraction of Zn^2+^ (L20C20Z2S1.5 with about 1.3 wt % of ZnCl_2_) achieved a notable tensile strength and Young modulus compared to the precursor without Zn^2+^ (L20C20S1.5). The tensile strength and toughness of L20C20Z2S1.5 (23.1 MPa and 44.6 MJ·m^−3^, respectively) reached the same level as that of C40S1.5 with a pure CB filler, and the Young modulus of the former (11.7 MPa) was even higher, as shown in Table 3. Meanwhile, as the Zn^2+^ dosage increased, the dispersion of lignin in rubber matrix was promoted (Figure S2, Supplementary Materials), which enhanced the interfacial interaction between lignin and the rubber matrix. As depicted in Figure 3a and Table 3, increasing the Zn^2+^ content caused a further increase in the strength, modulus and hardness with little sacrifice in the toughness and elasticity, suggesting a significant reinforcement effect of coordination sacrificial bonds on the mechanical performance. The tensile properties of pure NBR elastomers L0C0S1.5 and L0C0Z10S1.5 without fillers were also tested, as shown in Figure S3 and Table S2 (Supplementary Materials). The sample L0C0Z10S1.5 had a higher tensile strength and Young modulus than L0C0S1.5, also demonstrating that the physical crosslinking caused by the coordination sacrificial bonds in the rubber matrix had a significant reinforcement effect. Reducing the sulfur addition led to an improvement in the elongation at break but depressed the strength, as shown in Figure 3b. Interestingly, the tensile behaviors of L20C20S1.5 and L20C20Z4S0.5 were almost the same, indicating that the coordination bonds partially played a similar role as the sulfur bonds on endowing the mechanical performance. The difference was that the coordination bonds were non-permanent and could be dynamically fractured and reconstructed. The mechanical performance of lignin/CB/NBR elastomers can be manipulated by adjusting the proportion of non-permanent coordination sacrificial bonds and permanent covalent bonds.

### 3.4. Dynamic Features of Lignin/CB/NBR Elastomers

**Figure 4:**
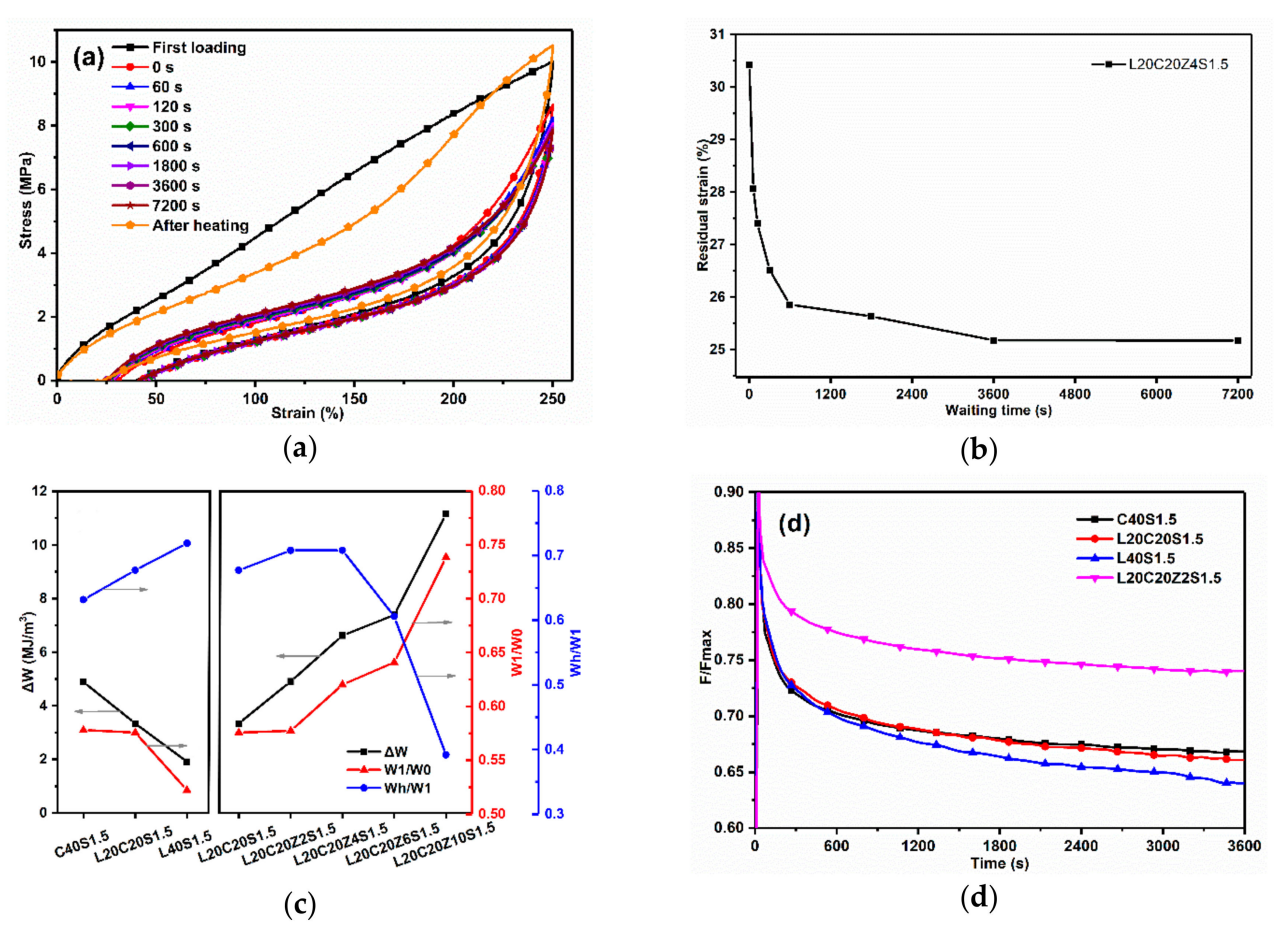
(**a**) Tensile loading-unloading curves for L20C20Z4S1.5; (**b**) dependence of residual strain for L20C20Z4S1.5 versus waiting time; (**c**) dependence of *W*_1_/*W*_0_, Δ*W* and *W*_h_/*W*_1_ for NBR elastomers with different Zn^2+^contents; (**d**) stress relaxation of lignin/CB/NBR elastomers at 200% strain.

Introducing Zn^2+^ into the lignin/CB/NBR elastomers produced a relatively strong coordination dynamic network. To reveal the deformation mechanism in the presence of Zn-based coordination bonds, hysteresis tests were carried out with a fixed strain of 250%. All samples exhibited similar hysteresis behavior, as shown in Figure S4 (Supplementary Materials). From the hysteresis curves of the sample L20C20Z4S1.5 (Figure 4a), one can see the energy dissipated in the first hysteresis cycle. After the first unloading, the sample left an obvious residual strain. The hysteresis loss and residual strain were mainly induced by the fracture of metal coordination bonds and rubber-filler network [18]. Subsequent loading-unloading cycles gave similar hysteresis loss. When the sample was relaxed for a certain time at 25 °C after a loading-unloading cycle, the residual strain decreased with an increasing relaxation time as shown in Figure 4b. Nevertheless, the tensile curve could not recover to its original one even after a long relaxation time (7200 s). This was because the temporary re-formation of Zn^2+^-based coordination bonds hindered the elastic recovery of rubber chains [16,17]. The tensile loading-unloading curves of samples without ZnCl_2_ (Figure S4a–c, Supplementary Materials) also exhibited similar characteristics. This was because the fracture of a rubber-filler network and the friction between rubber chain segments and fillers also retarded the elastic recovery of rubber chains. After relaxing for 40 min at 80 °C most of the temporarily re-formed coordination bonds broke and the covalent network recovered to its original state, resulting in nearly a complete disappearance of residual strain. Upon cooling, the coordination bonds re-formed again to the original equilibrium state of the covalent network, giving a mostly recovered stress-strain curve and hysteresis loss that were close to the first loading-unloading cycle (Figure 4a).

To further evaluate the energy dissipation of sacrificial bonds, the dependences of the hysteresis ratio *W*_1_/*W*_0_ and hysteresis difference Δ*W (W*_1_ − *W*_2_*)* on the ZnCl_2_ dosage are depicted in Figure 4c. The values of *W*_1_/*W*_0_ and *ΔW* increased monotonically with the ZnCl_2_ content, intuitively revealing a higher energy dissipation induced by the dynamic fracture of more coordination bonds. When adding 10 phr ZnCl_2_ into the elastomer (sample L20C20Z10S1.5), Δ*W* increased by about 2.7 times compared with that of L20C20S1.5. The recovery of the coordination sacrificial bonds was qualitatively examined by the hysteresis ratio *W*_h_/*W*_1_, also shown in Figure 4c. Increasing the ZnCl_2_ content led to a smaller *W*_h_/*W*_1_ value, suggesting a weaker hysteresis recovery. This was attributed to the principle of “strong means slow” [24]. A high ZnCl_2_ content led to more Zn-based bonds remaining unrecovered after healing at 80 °C for 30 min, thus exerting a stronger restriction on the elastic contraction of the primary chains.

To analyze the restriction effect of Zn-based bonds on the rubber chains, the stress relaxation of NBR elastomers was performed at 25 °C, as shown in Figure 4d. Compared to C40S1.5 and L20C20S1.5, L40S1.5 released more force, verifying a poorer restriction effect of lignin on rubber chains. When ZnCl_2_ was added, the relaxation process for L20C20Z2S1.5 was much slower than L20C20S1.5, suggesting the stronger restriction of the metal coordination bonds. With the increase of time, the tensile stress for all elastomers gradually tended to stabilize due to the energy storage by the sulfur covalent network [25].

The above evidence proved that the lignin/CB/NBR elastomers with ZnCl_2_ were mainly composed of a dual-crosslinking network structure including a sulfur covalent network and a dynamic coordination sacrificial network. The deformation mechanism of the dual-network lignin/CB/NBR elastomer is proposed in Scheme 2. At small strains, the rubber chain segments slip and rearrange along the stretching orientation. Upon further stretching, the Zn-based coordination bonds gradually fracture and the rubber-lignin interactions break. The dynamic rupture of the transient coordination sacrificial network allows for the release of excess stress, leading to the local relaxation and orientation of the rubber chain segments, which promotes efficient energy dissipation and prevents stress concentration. Therefore, an improved strength and elastic modulus (Figure 3a) were achieved.

**Figure 5:**
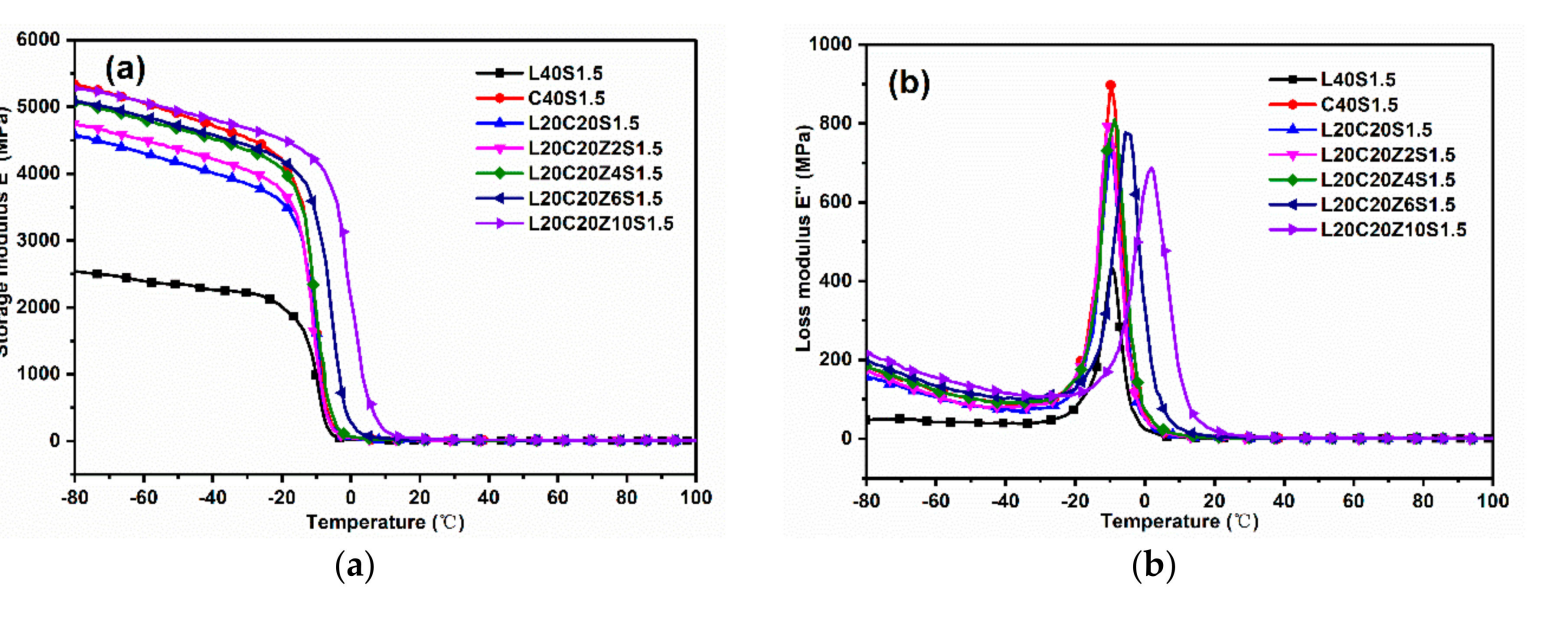

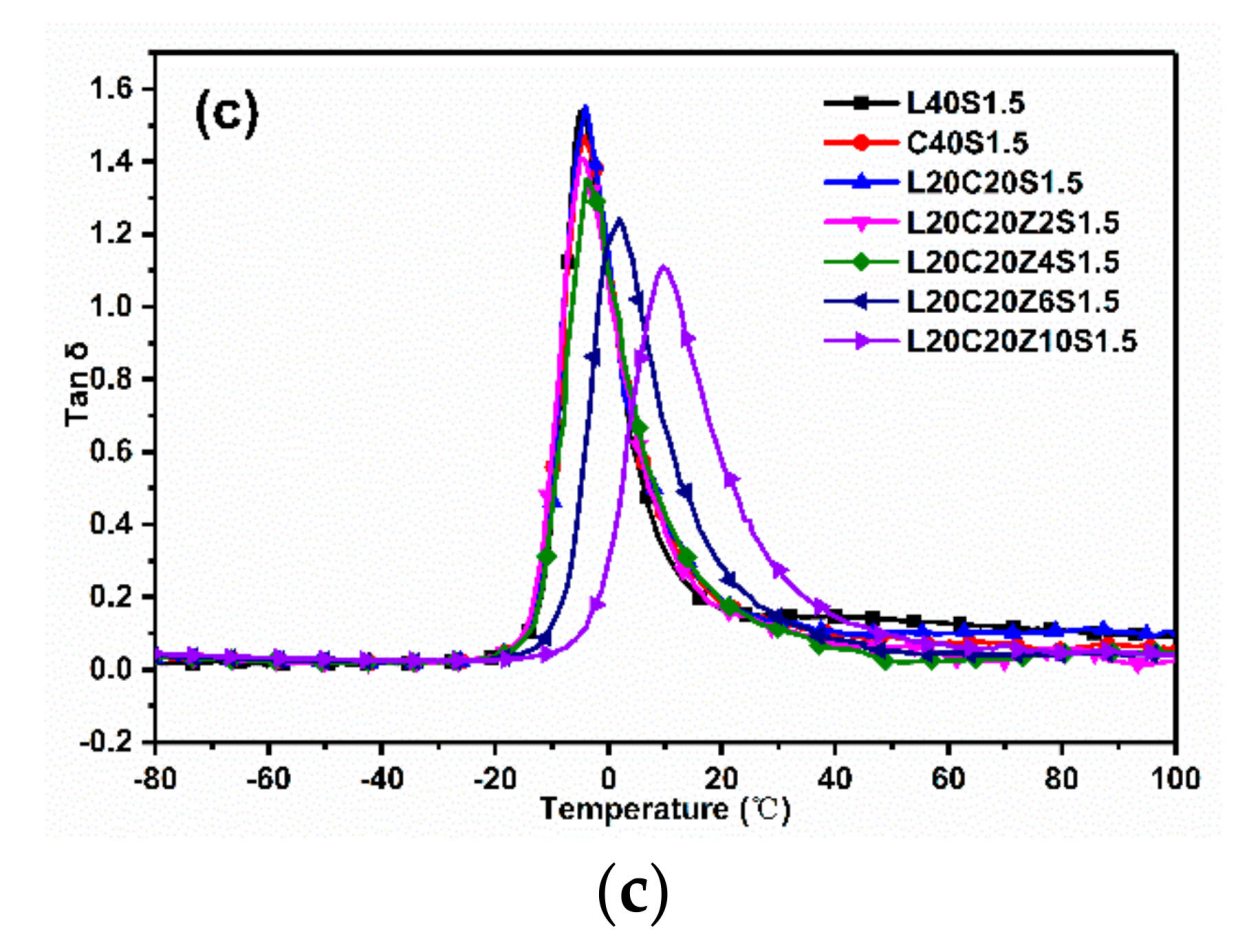
Dependence of (**a**) the storage modulus *E*′; (**b**) the loss modulus *E*″ and (**c**) tan δ versus temperature for NBR elastomers with different Zn^2+^ content.

The effects of coordination bonds on the thermomechanical properties of lignin/CB/NBR elastomers were studied by DMA analysis. Figure 5 presents the temperature dependences of the storage modulus (*E*′), loss modulus (*E*″) and loss factor (tan δ) for NBR elastomers with different Zn^2+^ contents. As shown in Figure 5a, when the temperature was lower than 20 °C, *E*′ of the elastomers decreased with the increasing lignin content (C40S1.5, C20L20S1.5 and L40S1.5) due to the weaker interaction between lignin and the rubber segments. With the incorporation of ZnCl_2_ into L20C20S1.5, the value of *E*′ at low temperature (less than 20 °C) increased gradually with the Zn^2+^ content. Simultaneously, the initial temperature of the rubbery plateau zone increased as well, caused by the binding of metal coordination bonds in the rubber elastomers. In addition, Figure 5b shows the value of *E*″ increased with the Zn^2+^ content at low temperature, attributed to the increased internal friction in the presence of metal coordination bonds. The glass transition temperatures (*T*_g_) of NBR elastomers determined from DMA analysis are listed in Table 4. As shown in Figure 5c, compared with the sample L20C20S1.5, the peak height decreased and the *T*_g_ value gradually increased with the increase of Zn^2+^ content, clearly suggesting the hindrance of coordination bonds on the segmental movement of rubber chains.

### 3.5. Thermal Behaviors of Lignin/CB/NBR Elastomers

**Figure 6:**
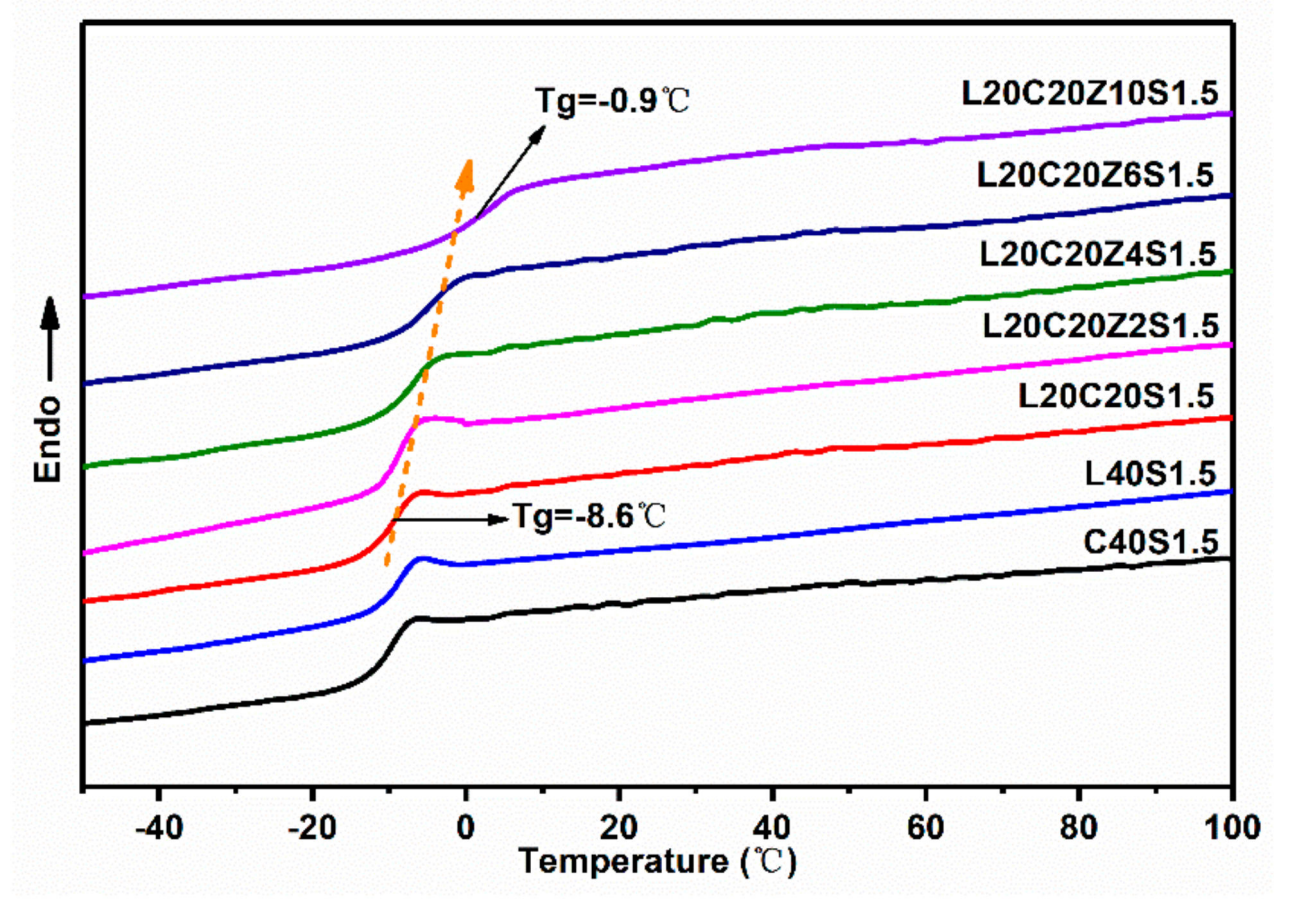
The DSC curves of NBR elastomers with different content of Zn^2+^.

The effect of the coordination of sacrificial bonds on the glass transition temperatures (*T*_g_) of lignin/CB/NBR elastomers was also studied by DSC, as presented in Figure 6 and Table 4. The *T*_g_ showed a slight increase when CB was substituted by lignin, attributing to the hydrogen-bonding interaction between lignin and NBR. As the Zn^2+^ content increased, more coordination bonds formed in the elastomers, which intensely limited the movement of NBR chain segments. As a result of the strong coordination interactions, the *T*_g_ of the elastomers increased from −8.6 °C without ZnCl_2_ (L20C20S1.5) to −0.9 °C with 10 phr ZnCl_2_ (L20C20Z10S1.5), consistent with the variation trend revealed by the DMA analysis.

**Figure 7:**
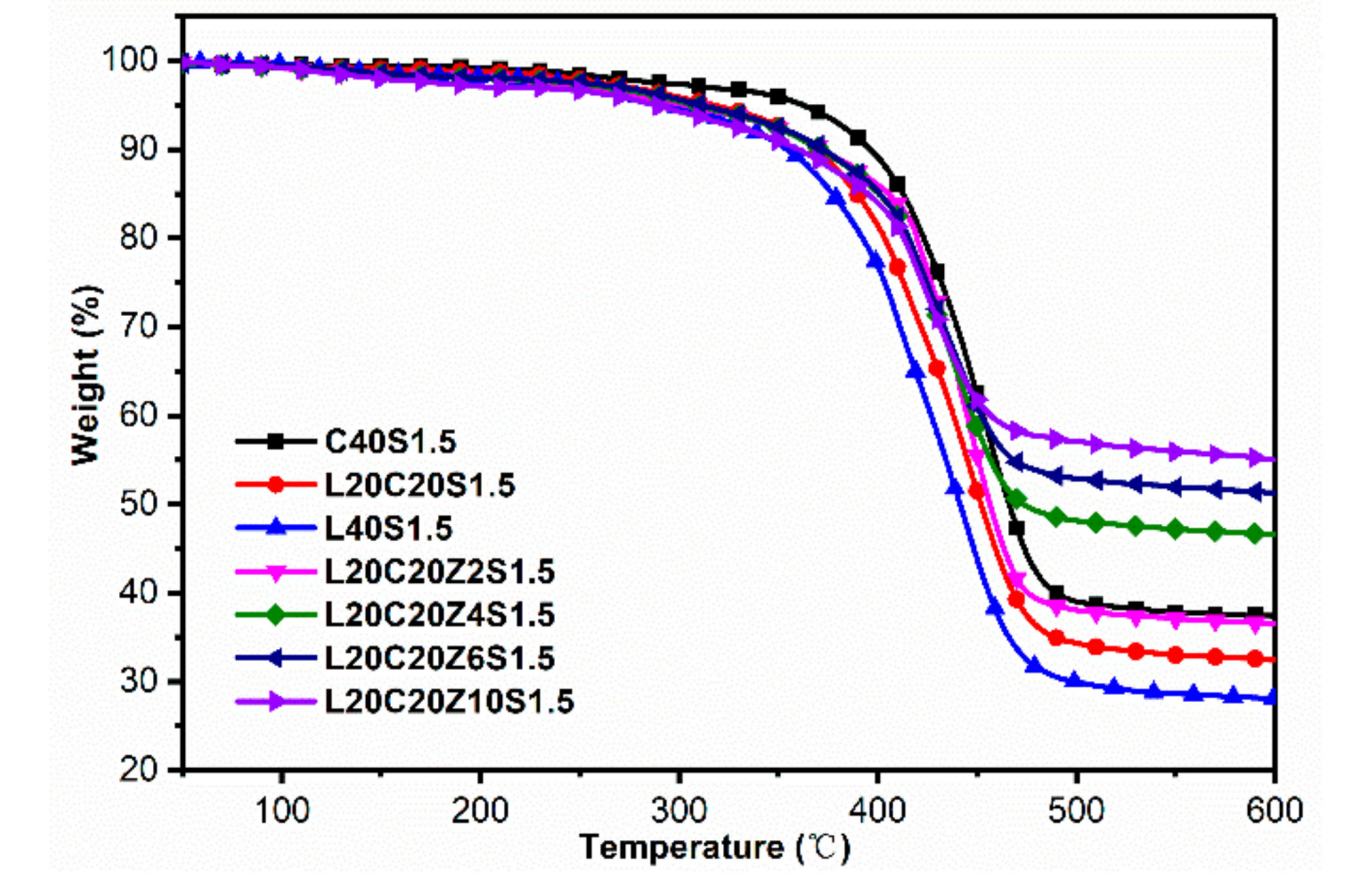
The TG curves for NBR elastomers with different contents of Zn^2+^.

TG analysis was performed to evaluate the thermal degradation of the elastomers, as shown in Figure 7. The characteristic degradation temperatures are listed in Table 4. All elastomers exhibited one main mass loss step. The sample filled with pure CB (C40S1.5) performed the best thermal stability. As lignin starts to decompose at about 200 °C [2], the initial decomposition temperature, *T*_10%_, of L40S1.5 (354.6 °C) was much lower than that of C40S1.5 (*T*_10%_ = 396.4 °C). The residue at 600 °C reduced as CB was replaced by lignin. When ZnCl_2_ was added into the lignin/CB/NBR elastomers, the initial decomposition temperature, *T*_10%_, and the temperature with 30% weight loss, *T*_30%_, were improved in a certain degree. Interestingly, the residue at 600 °C increased substantially with the Zn^2+^ content. The residue at 600 °C for sample L20C20Z10S1.5 reached 55.0%, much higher than that of C40S1.5 (37.4%) and L20C20S1.5 (32.4%). This might be caused by the flame retardance of the halogen and the carbonization effect of metal ions. Metal ions were found to facilitate the carbonization of lignin to form lignin-based carbon materials [26].

### 3.6. Oil Resistance of Lignin/CB/NBR Elastomers

**Figure 8:**
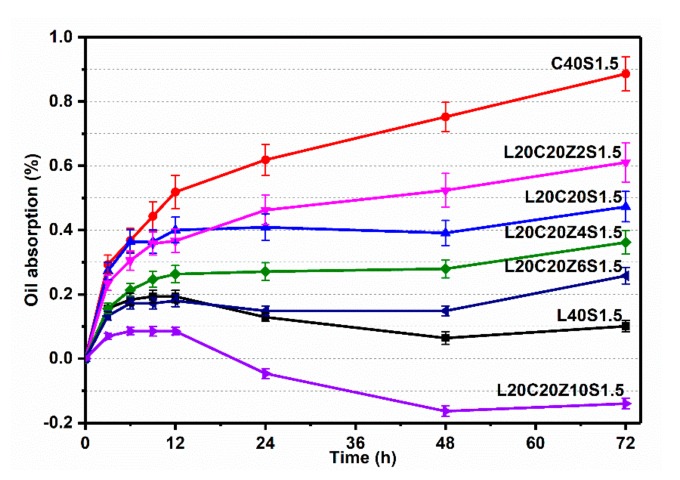
The high-temperature oil resistance of NBR elastomers at 100 °C.

NBR is usually used in oil resistant products. However, few data on the high-temperature oil resistance were reported for lignin/NBR elastomers. Here, the high-temperature oil resistance of lignin-filled NBR elastomers was studied by immersing the rubber specimens in hypoid gear oil at 100 °C for 72 h. As shown in Figure 8, the lignin-filled NBR elastomer (L40S1.5) had a much better oil resistance at 100 °C than the CB-filled NBR (C40S1.5). This was benefited by the large number of polar groups in the lignin molecules such as hydroxyls, which are more oleophobic than CB in the nonpolar oil. The oil resistance of sample L20C20S1.5 was in between the samples L40S1.5 and C40S1.5. When ZnCl_2_ was added into the elastomer of L20C20S1.5, as the content of Zn^2+^ increased, the metal coordination effect intensified and the crosslinking density of the rubber elastomer increased, which hindered the oil permeation into the rubber. Therefore, the oil absorption of the elastomers was suppressed with the rising Zn^2+^ content. The results showed that the high-temperature oil resistance of the NBR elastomers was improved by the incorporation of lignin and metal coordination bonds. It is worth noting that the oil absorption of L20C20Z10S1.5 slightly decreased between 12 and 48 h. This might be induced by the water absorption of ZnCl_2_. Even after the drying process, the sample L20C20Z10S1.5 could not be dehydrated completely and still kept some residual water. When immersed in oil at 100 °C, the residual water was squeezed out of the samples by oil, which created the illusion of a drop in oil absorption.

## 4. Conclusions

In conclusion, we have developed lignin/CB/NBR elastomers with a high strength, excellent elasticity and good energy dissipation by constructing a dual-crosslinking network consisting of the sulfur covalent bonds and Zn-based dynamic coordination sacrificial bonds through a conventional rubber compounding process. Lignin was not only used for the substitution of half the mass of CB in the NBR elastomer but also served as natural ligands for the Zn-based coordination bonds, which endowed a significant synergistic coordination enhancement effect. By adjusting the proportion of non-permanent coordination bonds and permanent covalent bonds, the mechanical performance of the lignin/CB/NBR elastomers can be manipulated. Increasing the Zn^2+^ content induced a higher energy dissipation through the dynamic rupture of Zn-based coordination bonds, leading to a significant improvement in the tensile strength, hardness and modulus of the elastomers with little sacrifice in the toughness and elasticity. Lignin/CB/NBR elastomers with a higher strength and modulus than CB-filled elastomers were obtained. The coordination bonds increased the glass transition temperature and improved the thermal stability of NBR elastomers. The high-temperature oil resistance of the NBR elastomers was also improved by the incorporation of lignin and metal coordination bonds. This work marks a new method for the preparation of high-performance and green lignin/rubber elastomers.

## Figures and Tables

**Table 1:** The formulations (phr) of lignin/CB/NBR elastomers ^1^.

Sample	Lignin	CB	ZnCl_2_	S
L0C0S1.5	0	0	0	1.5
L0C0Z10S1.5	0	0	10	1.5
C40S1.5	0	40	0	1.5
L20C20S1.5	20	20	0	1.5
L40S1.5	40	0	0	1.5
L20C20Z2S1.5	20	20	2	1.5
L20C20Z4S1.5	20	20	4	1.5
L20C20Z6S1.5	20	20	6	1.5
L20C20Z10S1.5	20	20	10	1.5
L20C20Z4S1.0	20	20	4	1.0
L20C20Z4S0.5	20	20	4	0.5

^1^ Other rubber ingredients were fixed as (phr): NBR 100, ZnO 5, SA 2, accelerator CZ 1.5, accelerator DM 0.5.

**Table 2:** The curing parameters ^1^ and crosslinking density of the elastomers.

Sample	*T*_S_ (min)	*T*_90_ (min)	*M*_L_ (dN.m)	*M*_H_ (dN.m)	Δ*M* (dN.m)	CRI (min^−1^)	*μ* (×10^−4^ mol/c
C40S1.5	0.73	15.02	1.57	17.26	15.69	6.99	3.28
L20C20S1.5	1.98	17.32	1.11	15.78	14.67	6.52	1.80
L40S1.5	2.32	18.55	0.66	13.19	12.53	6.16	1.33
L20C20Z2S1.5	1.22	9.96	1.17	20.61	19.44	11.44	2.18
L20C20Z4S1.5	1.20	9.78	1.35	22.18	20.85	11.64	2.67
L20C20Z6S1.5	1.32	9.50	2.25	23.41	21.16	12.21	2.93
L20C20Z10S1.5	1.12	8.08	2.88	26.42	23.54	14.35	3.36
L20C20Z4S1.0	1.47	9.97	1.41	17.93	16.18	11.75	1.35
L20C20Z4S0.5	2.02	12.98	1.65	12.67	11.02	9.12	0.43

*T*_S_: Scorch time; *T*_90_: Optimum cure time; *M*_L_: The minimum torque; *M*_H_: The maximum torque; Δ*M*: The difference between maximum torque and minimum torque; CRI: Curing rate index, CRI = 100/(*T*_90_ − *T*_S_). ^1^ *μ*: Crosslinking density.

**Table 3:** The mechanical properties of NBR elastomers.

**Sample**	**Elongation at Break (%)**	**Tensile Strength (MPa)**	**Young Modulus (MPa)**	**Energy Dissipation (MJ·m^−3^)**	**Elastic Recovery (%)**	**Hardness (Shore A)**
C40S1.5	408 (±11)	22.6 (±0.7)	9.3 (±0.2)	41.4	99.3 (±0.2)	67
L20C20S1.5	560 (±9)	19.4 (±0.4)	9.0 (±0.1)	44.0	98.9 (±0.1)	65
L40S1.5	702 (±23)	12.8 (±0.2)	7.8 (±0.2)	33.8	98.7 (±0.2)	63
L20C20Z2S1.5	418 (±6)	23.1 (±0.1)	11.7 (±0.3)	44.6	98.9 (±0.2)	70
L20C20Z4S1.5	326 (±5)	21.7 (±0.4)	12.1 (±0.2)	35.3	99.0 (±0.3)	71
L20C20Z6S1.5	336 (±5)	24.2 (±0.7)	14.7 (±0.3)	39.1	98.9 (±0.1)	73
L20C20Z10S1.5	332 (±16)	25.9 (±0.4)	13.4 (±0.4)	41.0	98.9 (±0.1)	75
L20C20Z4S1.0	442 (±15)	18.8 (±0.6)	9.8 (±0.1)	37.3	99.2 (±0.2)	69
L20C20Z4S0.5	579 (±8)	19.5 (±0.3)	9.2 (±0.2)	46.3	99.2 (±0.3)	66

**Table 4:** The glass transition temperatures and TG characteristics of the elastomers.

Sample	*T*_g_ (°C) from DSC	*T*_g_ (°C) from DMA	*T*_10%_^1^ (°C)	*T*_30%_^1^ (°C)	*T*_50%_^1^ (°C)	Residue at 600 °C (%)
C40S1.5	−10.0	−4.4	396.4	439.9	466.1	37.4
L20C20S1.5	−8.6	−4.1	368.6	421.8	452.0	32.4
L40S1.5	−8.5	−4.7	354.6	411.3	441.4	28.1
L20C20Z2S1.5	−8.4	−4.0	372.2	433.4	456.4	36.5
L20C20Z4S1.5	−5.9	−4.0	371.2	432.1	473.3	46.6
L20C20Z6S1.5	−4.3	1.9	373.0	433.5	--	51.3
L20C20Z10S1.5	−0.9	9.5	360.2	431.5	--	55.0

^1^*T*_10%_, *T*_30%_, *T*_50%_: Temperature corresponds to 10%, 30% and 50% mass loss, respectively; *T*_10%_ was regarded as the initial decomposition temperature.

## Supplementary Materials

The following are available online at http://www.mdpi.com/2073-4360/10/9/1033/s1, Figure S1: Curing curves of NBR elastomers L0C0S1.5 and L0C0Z10S1.5; Figure S2: The SEM photographs obtained from the fracture surface of lignin/CB/NBR elastomers: (a) L40S1.5; (b) L20C20S1.5; (c) C40S1.5; (d) L20C20Z2S1.5; (e) L20C20Z4S1.5; (f) L20C20Z6S1.5 and (g) L20C20Z10S1.5; Figure S3: The engineering stress-strain curves of NBR elastomers L0C0S1.5 and L0C0Z10S1.5; Figure S4: Tensile loading-unloading curves of (a) C40S1.5; (b) L40S1.5; (c) L20C20S1.5; (d) L20C20Z2S1.5; (e) L20C20Z6S1.5; (f) L20C20Z10S1.5; (g) L20C20Z4S0.5 and (h) L20C20Z4S1.0; Table S1: The curing parameters of NBR elastomers L0C0S1.5 and L0C0Z10S1.5. Table S2: The mechanical properties of NBR elastomers L0C0S1.5 and L0C0Z10S1.5.
