# Supplementary material for: Bioinspired Engineering towards Tailoring Advanced Lignin/Rubber Elastomers
Source: Polymers (Basel). 2018 Sep 18;10(9):1033. doi: 10.3390/polym10091033 (PMC6403759; doi:10.3390/polym10091033)
Supplement: Supplementary file 1 [file polymers-10-01033-s001.pdf]

Supplementary Information

Bioinspired engineering towards tailoring advanced lignin/rubber elastomers

Haixu Wang [1], Weifeng Liu [1,*] Jinhao Huang [1], Dongjie Yang [1] and Xueqing Qiu [1,2,*]

[1] School of Chemistry and Chemical Engineering, South China University of Technology, Guangzhou, 510640, China

[2] State Key Laboratory of Pulp and Paper Engineering, South China University of Technology, Guangzhou, 510640, China

* Correspondence: weifengliu@scut.edu.cn (W. L.); cexqqiu@scut.edu.cn (X. Q.); Tel.: +86-020-87114722

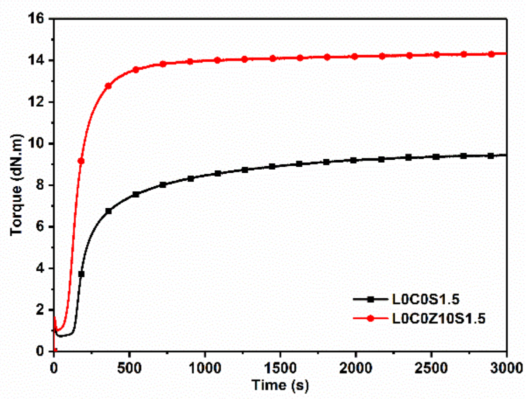

Figure S1. Curing curves of NBR elastomers L0C0S1.5 and L0C0Z10S1.5.

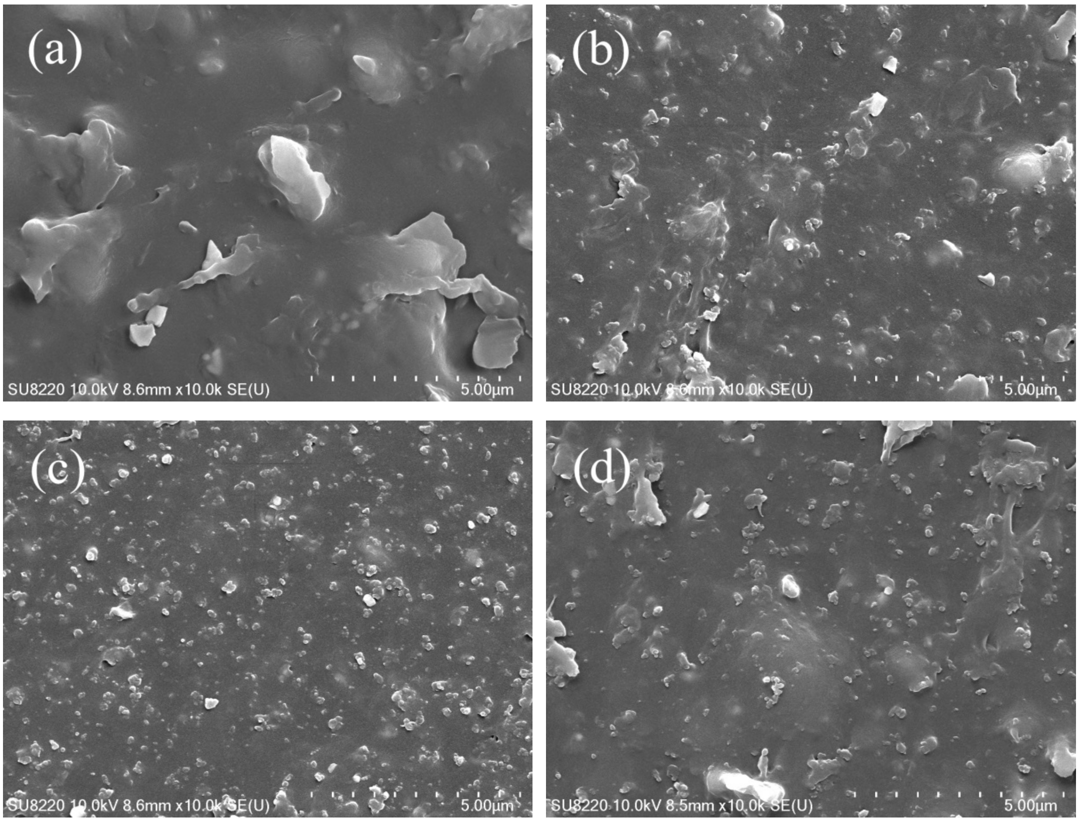

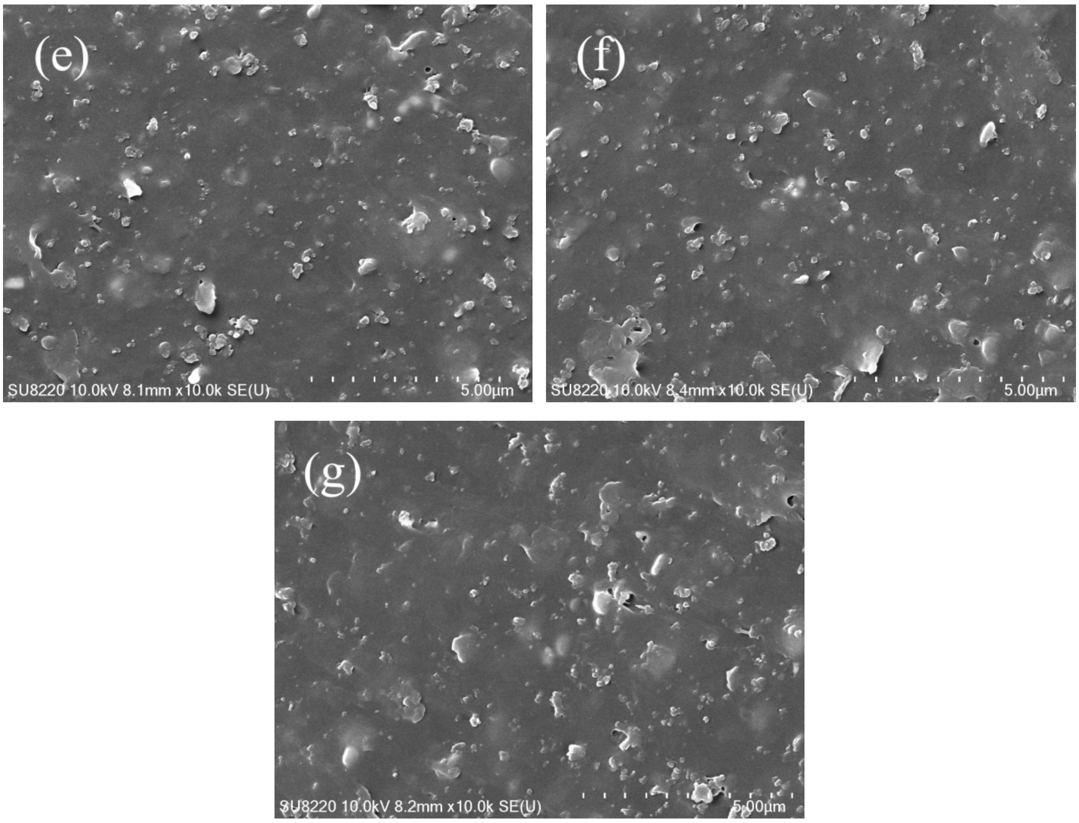

Figure S2. The SEM photographs obtained from the fracture surface of lignin/CB/NBR elastomers: (**a**) L40S1.5; (**b**) L20C20S1.5; (**c**) C40S1.5; (**d**) L20C20Z2S1.5; (**e**) L20C20Z4S1.5; (**f**) L20C20Z6S1.5 and (**g**) L20C20Z10S1.5.

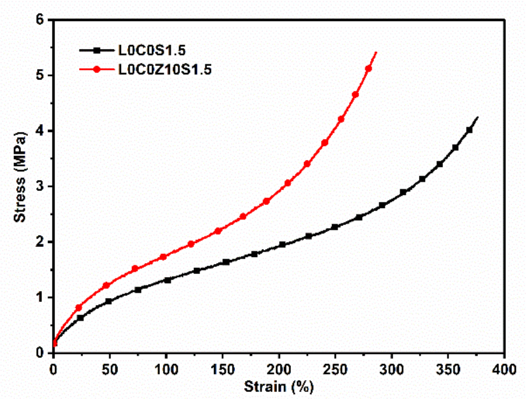

Figure S3. The engineering stress-strain curves of NBR elastomers L0C0S1.5 and L0C0Z10S1.5.

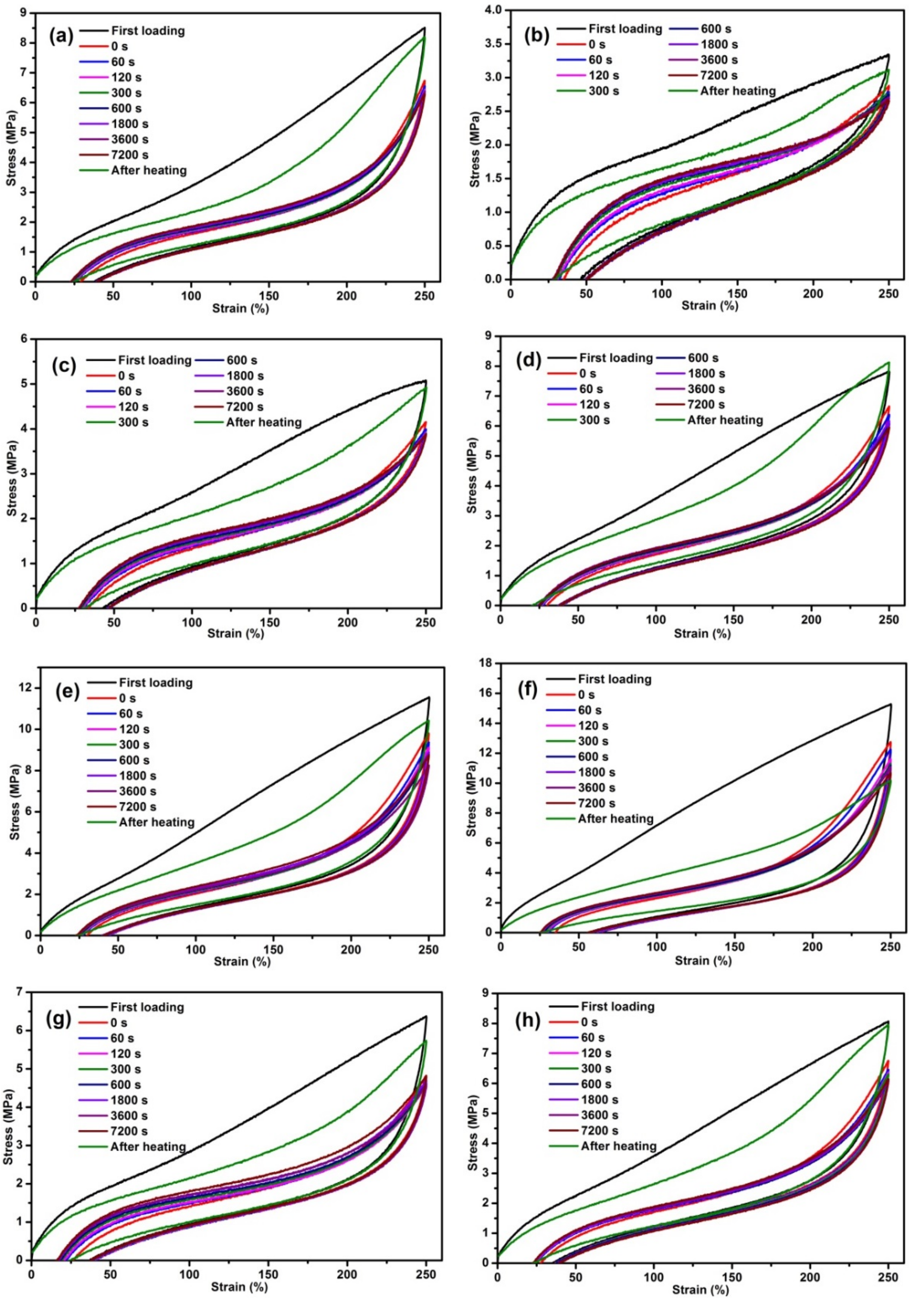

Figure S4. Tensile loading-unloading curves of (**a**) C40S1.5; (**b**) L40S1.5; (**c**) L20C20S1.5; (**d**) L20C20Z2S1.5; (**e**) L20C20Z6S1.5; (**f**) L20C20Z10S1.5; (**g**) L20C20Z4S0.5 and (**h**) L20C20Z4S1.0.

Table S1. The curing parameters[1] of NBR elastomers L0C0S1.5 and L0C0Z10S1.5.

Sample	T_S (min)	T_{90} (min)	M_L (dN.m)	M_H (dN.m)	ΔM (dN.m)	CRI (min^{-1})
L0C0S1.5	2.68	18.13	0.61	9.45	8.84	6.47
L0C0Z10S1.5	1.70	6.58	0.88	14.36	13.48	20.49

[1] T_S: scorch time; T_{90}: optimum cure time; M_L: the minimum torque; M_H: the maximum torque; ΔM: the difference between maximum torque and minimum torque; CRI: curing rate index, CRI = 100 / (T_{90}-T_S).

Table S2. The mechanical properties of NBR elastomers L0C0S1.5 and L0C0Z10S1.5.

Sample	Elongation at break (%)	Tensile strength (MPa)	Young modulus (MPa)	Energy dissipation (MJ·m^{-3})	Elastic recovery (%)	Hardness (Shore A)
L0C0S1.5	367(±15)	4.1(±0.2)	6.0(±0.2)	7.4	99.5(±0.3)	54
L0C0Z10S1.5	282(±10)	5.2(±0.2)	7.2(±0.3)	6.8	99.3(±0.2)	54
